# An updated systematic review of buffalo nutritional requirements (2000–2024)

**DOI:** 10.1007/s11250-026-05311-3

**Published:** 2026-09-04

**Authors:** Jarbas Miguel da Silva Junior

**Affiliations:** State University of Western Paraná, Marechal Cândido Rondon, Paraná, 85960000 Brazil

**Keywords:** Buffalo nutrition, Nitrogen requirements, Net energy requirements, Mineral requirements

## Abstract

Buffaloes (*Bubalus bubalis*) are large ruminants with a superior ability to digest and utilize low-quality forages, producing milk and meat of higher quality compared to cattle. Despite their importance in animal production, buffaloes remain the focus of relatively few studies aimed at defining their nutritional requirements, which often results in inadequate diet formulation and management. There, this study aimed to conduct a systematic review of the literature on buffalo nutritional requirements, considering only open-access papers published in English and in indexed journals. A total of 58 studies met the inclusion criteria. Most of them focused on dairy buffaloes, particularly on the evaluation of crude protein, energy levels, and fiber content in the diet. Mineral nutrition was addressed in eight studies; however, in general the evaluated levels showed no significant effects. In contrast, very few or no studies were found concerning beef buffaloes, growing animals, reproduction, or other production stages. These findings highlight a critical gap in the literature and reinforce the urgent need to direct more research efforts and resources toward establishing species-specific nutritional requirements, particularly for beef buffaloes.

## Introduction

According to the Food and Agriculture Organization of the United Nations, there are approximately 205 million buffaloes (*Bubalus bubalis*) worldwide, with nearly 98% of the population concentrated in Asia (FAO 2025). Buffaloes are large ruminants primarily used for milk and meat production, though they are also employed as draft animals (Marai and Haeeb 2010; Mota-Rojas et al. 2021). In addition, the buffalo production sector has experienced sustained global growth. The global buffalo meat sector was valued at approximately USD 3.23 billion in 2024 (TechSci Research 2025), while the buffalo milk sector is projected to increase by more than USD 21.87 billion between 2024 and 2029 (Market Research Reports 2025). This expanding production chain reinforces the need for updated knowledge on buffalo nutrition to support sustainable and efficient production systems.

Nutritionally, buffaloes are frequently treated as being analogous to cattle (*Bos taurus*). However, they constitute a distinct species with unique physiological and productive traits. For instance, they exhibit differences in body structure and growth patterns (tissue deposition) (Glatz 2022), differences in ruminal microbial composition, which may contribute to their greater capacity to digest and utilize low-quality forages and diets rich in structural carbohydrates (Bartocci et al. 2006; Chanthakhoun et al. 2012b; Iqbal et al. 2018; Azmi et al. 2021). Additionally, buffaloes have a longer gestation period (305 vs. 285 days), higher milk fat content (7% vs. 3.5%), and distinct carcass characteristics and meat composition compared with cattle (Jainudeen 2004; Sindhu and Arora 2011; Purohit 2016; Mollica et al. 2021; Andrade et al. 2023; Shazly et al. 2023). These factors, by themselves, have a significant impact on the animals’ nutritional requirements (Rotta et al. 2023).

Unlike cattle, which have well-established feeding standards (e.g., NRC/NASEM), buffaloes still lack widely adopted species-specific feeding standards because only a limited number of studies have directly evaluated their nutrient requirements. As a result, most nutritional recommendations for buffaloes are extrapolated from cattle data. This gap poses a substantial challenge for nutritionists and producers, as nutrition plays a central role in buffalo farming, directly influencing growth performance, reproductive efficiency, milk composition, and overall herd health (Iqbal et al. 2018; Glatz 2022; Rotta et al. 2023). Also, feed represents the largest component of production costs in buffalo systems, underscoring the need for adequate nutrient supply to ensure both productive efficiency and economic sustainability (Bartocci et al. 2006; Rotta et al. 2023). Moreover, the species’ ability to utilize fibrous and low-quality forages does not negate the importance of balanced diets, particularly in intensive and semi-intensive systems where suboptimal nutrition can markedly constrain performance. Consequently, the lack of species-specific information may result in nutrient under- or over-supply, with detrimental effects on animal performance, product quality, and farm profitability (Paul 2011; Stasio and Brugiapaglia 2021; Javed et al. 2022; Pires et al. 2022; Ponnampalam et al. 2024).

Thus, given this critical knowledge gap, the aim of this study was to conduct a systematic review of buffalo nutritional requirements, consolidating currently available research published between 2000 and 2024, and providing evidence-based guidance to support future studies and improve feeding strategies for this economically important species.

## Materials and methods

This research consisted of a systematic review on buffalo nutrition, specifically focusing on advances in nutritional requirements studies conducted over the last 25 years (2000–2024). The review included studies evaluating nutritional requirements related to meat and milk production in buffaloes. The review methodology was developed following the recommendations of the PRISMA 2020 statement for systematic reviews. Additionally, a review protocol was not prospectively registered because no formal meta-analysis was planned.

For the purposes of this review, nutritional requirements were defined operationally as the quantitative amounts of nutrients necessary to support maintenance, growth, reproduction, lactation, or work in buffaloes under specific physiological and environmental conditions. This definition includes classical requirement-estimation studies, in which graded nutrient levels are used to derive responses in performance or metabolism, as well as nutrition-response studies that provide indirect evidence relevant to requirement estimation, such as intake responses, digestibility outcomes, metabolic indicators, and production responses under different nutritional interventions.

### Inclusion and exclusion criteria

Articles were included if they: (1) were published in English between 2000 and 2024; (2) appeared in indexed journals; and (3) contained relevant information related to buffalo nutritional requirements.

The following were excluded: books, book chapters, conference proceedings, technical manuals, and abstracts published in conference proceedings.

### Selection of databases

The search was conducted twice: from February 17 to 29 (first round) and from July 25 to August 8 (second round), 2025, in the following databases: ScienceDirect (Elsevier), Springer Nature Link, CABI Digital Library, and Google Scholar. These platforms were selected for their accessibility, broad coverage of animal and nutritional sciences, and international relevance.

A preliminary exploratory search was carried out to evaluate the coverage of each platform before the final selection. This preliminary search used the following combination of terms: “Buffalo” AND “Animal” AND “Nutrition” AND “Nutritional” AND “Requirements”.

### Search strategy

The final search strategy used the following Boolean combination of terms in all databases: “Buffalo” AND “Requirements” AND (“Dry Matter Intake” OR “Nitrogen” OR “Net Energy” OR “Fiber” OR “Mineral”) AND (“Beef” OR “Milk” OR “Dairy”). When possible, filters were applied to limit results to peer-reviewed research papers, reviews, or technical notes, and to exclude other document types (e.g., theses, books).

All retrieved records were exported or manually copied into a spreadsheet, where duplicates were identified and removed.

Then, titles and abstracts were screened by a single author to determine eligibility according to the predefined inclusion criteria. All screening decisions were subsequently checked for consistency during the full-text evaluation process. For studies with unclear eligibility based on abstracts, the full text was read before a final decision was made.

Due to the limited number of papers retrieved, studies that evaluated nutrient inclusion levels (e.g., protein or energy supplementation) or the importance of specific minerals in buffalo diets were also included if they met the other inclusion criteria.

### Data extraction and analysis

Data extraction was performed manually using a structured spreadsheet. For each eligible paper, the following information was collected: first author’s name and publication year, country of origin (where the study was conducted), animal breed and number of animals used, main aim of the study, and type of production system (meat or milk/dairy).

The extracted data were summarized through descriptive analysis (no statistical synthesis or meta-analysis was performed due to limitations related to the number of animals and breeds used, study objectives, methodological data collection procedures, and statistical analyses).

## Results

A total of 8,160 records were found on the Google Scholar platform, followed by ScienceDirect (5,643), Springer Nature Link (1,533), and the CABI Digital Library, which showed the lowest number of records (728) when the search term “Buffalo + Animal + Nutrition” was used (Table 1). These numbers represent the initial search outputs and are reported solely for transparency of the search process, not for comparison between databases or study quality.

**Table 1:** Number of records retrieved from the selected databases (ScienceDirect [Else-vier], Google Scholar, Springer Nature Link, and CABI Digital Library) using buffalo nutrition-related search terms, used as a criterion for platform selection

Used term	Number of records by platform			
	Google Scholar	ScienceDirect	Springer Nature	CABI Library
Buffalo + Animal + Nutrition	8,160	5,643	1,533	728
Buffalo + Nutritional + Requirements	3,450	2,785	913	585

The search term “Buffalo + Nutritional + Requirements” yielded the highest number of records in Google Scholar (3,450), followed by ScienceDirect (2,785), Springer Nature Link (913), and CABI Digital Library (585).

**Fig. 1:**
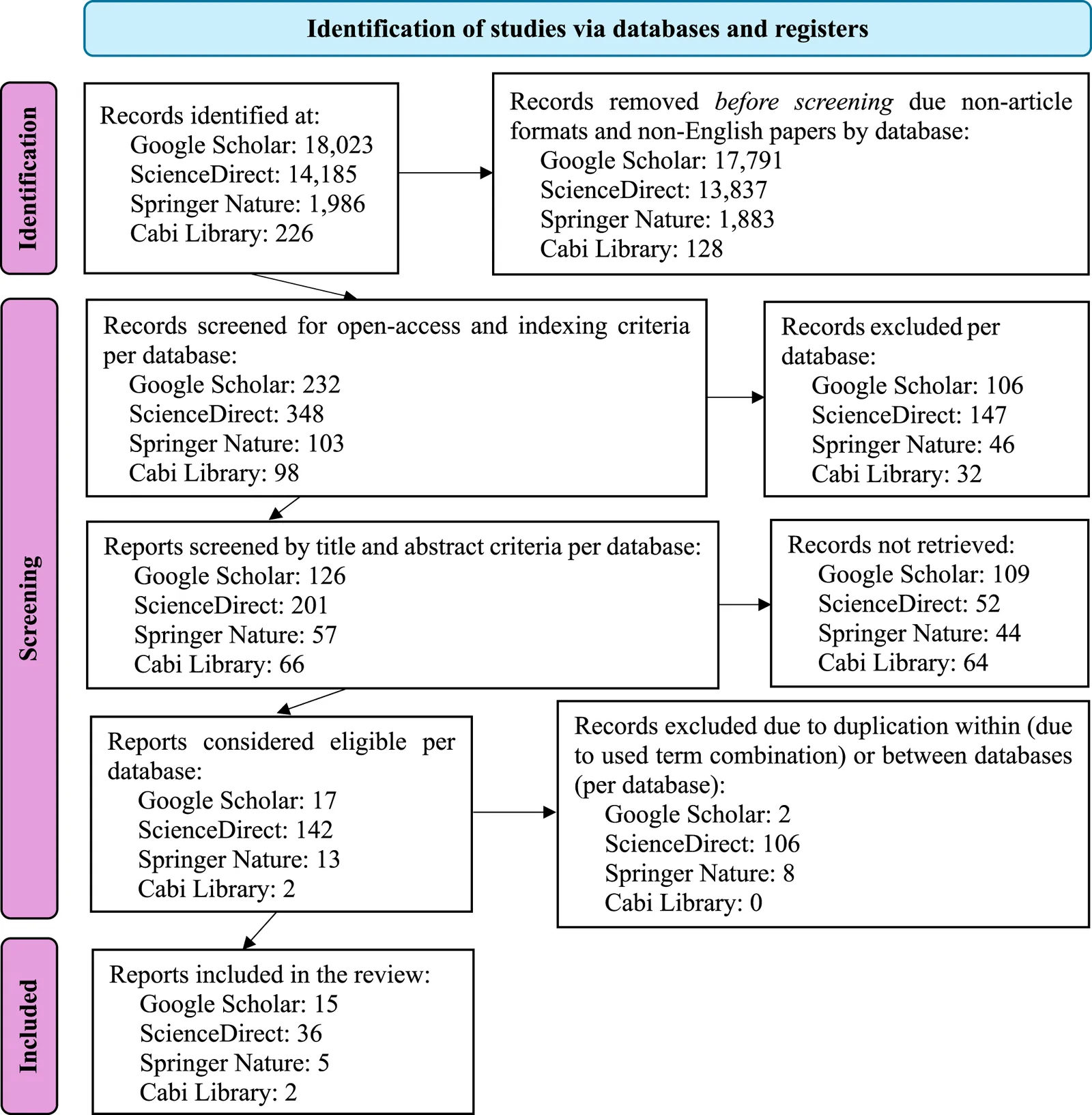
The PRISMA flow diagram depicts the systematic process of identifying, screening, assessing eligibility, and including studies in this review

The results of the systematic search are summarized in Fig. 1 and Table 2. Although Google Scholar returned the highest number of records (18,023), ScienceDirect yielded the greatest number of eligible studies identified after screening (36). It is important to note that, despite the use of targeted search terms, many retrieved records were unrelated to buffalo nutritional requirements and were excluded during screening. Searches conducted in the CABI Digital Library and Springer Nature Link retrieved only a limited number of papers; Springer Nature yielded five studies, whereas CABI yielded only two after the full screening process.

**Table 2:** Number of records and number of studies that met the inclusion criteria by search term (without removing duplicate papers across databases) in the selected databases (Google Scholar, ScienceDirect [Elsevier], CABI Digital Library, and Springer Nature Link), related to buffalo nutritional requirements for dry matter, crude protein, net energy, fiber, and minerals in meat- or milk/dairy-related experiments

Used term combination	Number of records by database							
	Google Scholar		ScienceDirect		CABI Digital		Springer Nature	
	Records	Criteria	Records	Criteria	Records	Criteria	Records	Criteria
Buffalo + Requirements + Dry Matter Intake	385	0	290	0	7	0	22	0
Buffalo + Requirements + Dry Matter Intake + Beef	280	0	117	0	6	0	12	0
Buffalo + Requirements + Dry Matter Intake + Milk	361	0	226	2	6	0	14	0
Buffalo + Requirements + Dry Matter Intake + Dairy	364	0	234	0	6	0	18	0
Buffalo + Requirements + Nitrogen	2,720	2	2,475	2	27	0	175	0
Buffalo + Requirements + Nitrogen + Beef	857	0	439	1	14	0	30	0
Buffalo + Requirements + Nitrogen + Milk	1,380	1	915	1	18	0	60	0
Buffalo + Requirements + Nitrogen + Dairy	1,380	0	846	2	14	0	52	0
Buffalo + Requirements + Net Energy	167	0	196	1	3	1	8	0
Buffalo + Requirements + Net Energy + Beef	80	0	65	1	3	0	2	0
Buffalo + Requirements + Net Energy + Milk	101	0	113	3	3	0	4	0
Buffalo + Requirements + Net Energy + Dairy	107	0	131	1	3	0	4	0
Buffalo + Requirements + Fiber	1,970	3	2,036	35	19	1	708	4
Buffalo + Requirements + Fiber + Beef	633	3	393	15	10	0	101	2
Buffalo + Requirements + Fiber + Milk	969	2	749	32	15	0	304	4
Buffalo + Requirements + Fiber + Dairy	946	0	679	28	13	0	254	3
Buffalo + Requirements + Mineral	2,160	2	2,005	9	24	0	103	0
Buffalo + Requirements + Mineral + Beef	693	1	440	2	9	0	19	0
Buffalo + Requirements + Mineral + Milk	1,250	1	934	2	14	0	47	0
Buffalo + Requirements + Mineral + Dairy	1,220	2	902	5	12	0	49	0

Searches including the terms “milk” or “dairy” produced more eligible studies than those combined with “beef,” reflecting the predominance of research conducted in dairy production systems. When the search terms were combined with “fiber”, the largest number of studies meeting the inclusion criteria was observed overall. Mineral requirements represented the topic with the highest number of relevant publications, whereas studies addressing dry matter (DM) intake (DMI), crude protein (CP), fiber, total digestible nutrients (TDN), nitrogen requirements, or energy requirements were scarce compared with mineral-related studies and overall topics and rarely met the inclusion criteria after full screening.

After the removal of duplicates and the application of all screening steps, a total of 58 studies were eligible and included in the final synthesis (Table 3). It was observed that none of the works directly evaluated the nutritional requirements of buffaloes; instead, they assessed levels of nutrient inclusion, their importance, or estimated the animals’ requirements based on dietary content and performance. It is also noteworthy that the majority of the papers focused on milk/dairy scenarios.

**Table 3:** List of papers meeting the final criteria and included in the systematic review on buffalo nutritional requirements

First author	Year	Title	Journal	Country	Animals
Verma	2000	Revival performance of growing buffalo calves fed urea molasses liquid diet and roughage as survival feed	Animal Feed Science and Technology	India	*N° of animals*: 8 *Animal breed used*: not specified *Production*: Growing buffalo calves *Aim*: Evaluate two levels of roughage combined with urea molasses liquid diet as survival feed
Tiwari	2001	Studies on blood biochemical constituents and rumen fermentation in growing buffalo calves fed ammoniated straw-based rations supplemented with different protein sources	Animal Feed Science and Technology	India	*N° of animals*: 18 *Animal breed used*: Murrah *Production*: Growing buffaloes *Aim*: Study the rumen fermentation and blood biochemical changes in growing buffalo calves fed urea-ammoniated straw based rations
Bartocci	2002	Characteristics of foodstuffs and diets, and the quanti-qualitative milk parameters of Mediterranean buffaloes bred in Italy using the intensive system: An estimate of the nutritional requirements of buffalo herds lactating or dry	Livestock Production Science	Italy	*N° of animals*: 1,945 *Animal breed used*: Mediterranean *Production*: Milk *Aim*: To evaluate the animals’ nutritional requirements based on data collected from 20 farms
Paul	2004	Effect of administration of an anaerobic gut fungus isolated from wild blue bull (*Boselaphus tragocamelus*) to buffaloes (*Bubalus bubalis*) on in vivo ruminal fermentations and digestion of nutrients	Animal Feed Science and Technology	India	*N° of animals*: 8 *Animal breed used*: not specified *Production*: Growing buffaloes *Aim*: To evaluate the effect of administration of an elite strain of anaerobic fungi on nutrient digestion, ruminal fermentation and microbial population
Bartocci	2006	The utilization of a high level energy/protein diet for lactating Mediterranean buffaloes: Intake capacity and effects on quanti–qualitative milk parameters	Livestock Production Science	Italy	*N° of animals*: 14 *Animal breed used*: Mediterranean *Production*: Milk *Aim*: To evaluate the voluntary intake and milk yield performance of buffalo cows in early lactation fed a high-energy and high-protein diet
Sarwar	2006	Influence of urea molasses treated wheat straw fermented with cattle manure on chemical composition and feeding value for growing buffalo calves	Livestock Scinece	Pakistan	*N° of animals*: 28 *Animal breed used*: not specified *Production*: Growing buffaloes *Aim*: To examine the chemical composition of urea-molasses treated wheat straw fermented with cattle manure and its feeding value for growing buffalo male calves
Aasif Shahzad	2008	Influence of altering dietary cation anion difference on milk yield and its composition by early lactating Nili Ravi buffaloes in summer	Livestock Science	Pakistan	*N° of animals*: 20 *Animal breed used*: Nili Ravi *Production*: Milk *Aim*: Test variations on dietary cation anion differences on dry matter intake, nutrient digestibility, nitrogen balance and milk yield
Naik	2009	Effect of ruminally protected fat on in vitro fermentation and apparent nutrient digestibility in buffaloes (*Bubalus bubalis*)	Animal Feed Science and Technology	India	*N° of animals*: 20 *Animal breed used*: not specified *Production*: Adult buffaloes *Aim*: To evaluate the effects of supplementation of calcium salts of long chain fatty acids as rumen inert fat on in vitro fermentation and apparent nutrient digestion
Sultan	2009	Effect of varying ruminally degradable to ruminally undegradable protein ratio on nutrient intake, digestibility and N metabolism in Nili Ravi buffalo calves (*Bubalus bubalis*)	Livestock Science	Pakistan	*N° of animals*: 4 *Animal breed used*: Nili-Ravi *Production*: Growing buffaloes *Aim*: To evaluate the influence of varying ruminally degradable protein to ruminally undegradable protein on DM intake, digestibility and nitrogen metabolism
Campanile	2010a	Protein nutrition and nitrogen balance in buffalo cows	CABI Reviews	Italy	This paper is a review article and therefore does not provide specific details regarding animals *Production*: Milk *Aim*: Discuss the protein metabolism
Campanile	2010b	Growth, metabolic status and ovarian function in buffalo (*Bubalus bubalis*) heifers fed a low energy or high energy diet	Animal Reproduction Science	Brazil	*N° of animals*: 12 *Animal breed used*: Murrah *Production*: Growth of Buffaloes heifers and their reproduction *Aim*: Evaluate the animals’ ability to maintain metabolic homeostasis under a low-energy diet
Khattab	2010	Effect of different additive sources on milk yield and composition of lactating buffaloes	Livestock Science	Egypt	*N° of animals*: 16 *Animal breed used*: not specified *Production*: Milk *Aim*: To study the effect of chemical, biological and natural additives either alone or combination on milk yield and composition
Paul	2011	Nutrient requirements of buffaloes	Brazilian Journal of Animal Science	India	This paper is a review article and therefore does not provide specific details regarding animals *Aim*: Discuss buffaloes’ nutritional requirements throughout a literature review
Gandra	2011	Productive performance, nutrient digestion and metabolism of Holstein (*Bos taurus*) and Nellore (*Bos taurus indicus*) cattle and Mediterranean Buffaloes (*Bubalis bubalis*) fed with corn-silage based diets	Livestock Science	Brazil	*N° of animals*: 10 *Animal breed used*: Mediterranean *Production*: Growing animals *Aim*: To evaluate the productive performance, nutrients digestion and metabolism of three different genetic groups fed with the same diet based on corn silage
Javaid	2011	Ruminal dynamics of *ad libitum* feeding in buffalo bulls receiving different level of rumen degradable protein	Livestock Science	Pakistan	*N° of animals*: 4 *Animal breed used*: Nili-Ravi *Production*: Growing animals *Aim*: to determine the influence of varying level of ruminally degradable protein (RDP) on dry matter intake, ruminal characteristics, digestibility, blood pH, blood urea nitrogen and nitrogen balance
Aasif Shahzad	2011	Effect of feeding different dietary protein and energy levels on the performance of 12-15-month-old buffalo calves	Tropical Animal Health and Production	Pakistan	*N° of animals*: 60 *Animal breed used*: Nili-Ravi *Production*: Growing animals *Aim*: To investigate the performance response of different dietary protein and energy levels
Tauqir	2011	Response of growing buffalo calves to various energy and protein concentrations	Livestock Science	Pakistan	*N° of animals*: 36 *Animal breed used*: Nili Ravi *Production*: Growth animals *Aim*: Evaluate the impact of crude protein and metabolizable energy variations on buffalo growth
Zia-ul-Hassan	2011	Nutrient utilization and milk yield response of early lactating Nili-Ravi buffaloes fed on urea-molasses treated wheat straw fermented with cattle manure	Livestock Science	Pakistan	*N° of animals*: 20 *Animal breed used*: Nili-Ravi *Production*: Milk *Aim*: to examine the influence of urea–molasses treated wheat straw fermented with cattle manure on nutrients intake and their digestibilities, milk yield and its composition
Chanthakhoun	2012b	Level of crude protein in concentrate supplements influenced rumen characteristics, microbial protein synthesis and digestibility in swamp buffaloes (*Bubalus bubalis*)	Livestock Science	Thailand	*N° of animals*: 4 *Animal breed used*: Thai Swamp *Production*: Growth and production animals *Aim*: Evaluate the impact of crude protein levels on rumen characteristics and nutrient digestibility
Kang	2012	Effects of energy level and *Leucaena leucocephala* leaf meal as a protein source on rumen fermentation efficiency and digestibility in swamp buffalo	Animal Feed Science and Technology	Thailand	*N° of animals*: 4 *Animal breed used*: not specified *Production*: Growing animals *Aim*: To investigate the effect of energy level with heated and unheated treatment on Leucaena leucocephala leaf meal on feed intake, nutrient digestibility, rumen fermentation and microbial population
Shelke	2012	Effect of feeding protected fat and proteins on milk production composition and nutrient utilization in Murrah buffaloes	Animal Feed Science and Technology	India	*N° of animals*: 18 *Animal breed used*: Murrah *Production*: Milk *Aim*: To investigate the effect of feeding protected fat and proteins on milk production
Garg	2013	Effects of feeding nutritionally balanced rations on animal productivity, feed conversion efficiency, feed nitrogen use efficiency, rumen microbial protein supply, parasitic load, immunity and enteric methane emissions of milking animals under field conditions	Animal Feed Science and Technology	India	*N° of animals*: 12,518 *Animal breed used*: Not specified *Production*: Milk *Aim*: Investigate the impact of providing a balanced diet on animal productivity
Hung	2013	Effects of Leucaena leaf pellet on bacterial diversity and microbial protein synthesis in swamp buffalo fed on rice straw	Livestock Science	Thailand	*N° of animals*: 4 *Animal breed used*: not specified *Production*: Growing animals *Aim*: To evaluate the effect of pelleted levels of leucaena leaf, urea and some binding agent on microbial synthesis and diversity
Deka	2014	Body condition, energy balance and immune status or periparturient Murrah buffaloes (*Bubalus bubalis*) supplemented with inorganic chromium	Biological Trace Elements Research	India	*N° of animals*: 24 *Animal breed used*: Murrah *Production*: Beef *Aim*: To test the dietary chromium supplementation improves energy balance and immune response in perpartum
Riaz	2014	Voluntary feed intake and digestibility of four domestic ruminant species as influenced by dietary constituents: a meta-analysis	Livestock Science	Switzerland	*This paper is a meta-analysis article and therefore does not provide specific details regarding animals* *Aim: Discuss buffaloes’ and other ruminants’ nutritional aspects*
Sabia	2014	Efficiency to reach age of puberty and behaviour of buffalo heifers (*Bubalus bubalis*) kept on pasture or in confinement	Animal	Italy	*N° of animals*: 32 *Animal breed used*: Mediterranean *Production*: Growth of heifer’s buffaloes *Aim*: Assess the effects of confinement or free-ranging buffalo heifers on their growth efficiency and attainment of puberty
Singh	2014	Effect of dietary protein on intake, nutrients utilization, nitrogen balance, blood metabolites, growth and puberty in growing Bhadawari buffalo (*Bubalus bubalis*) heifers	Tropical Animal Health and Production	India	*N° of animals*: 15 *Animal breed used*: Bhadawari *Production*: Growing heifers *Aim*: To evaluate the effect of protein level on nutrient utilization, nitrogen balance, growth rate, blood metabolites, and puberty
Deka	2015	Effect of additional chromium supplementation on health status, metabolic responses, and performance traits in periparturient Murrah buffaloes (*Bubalus bubalis*)	Biological Trace Elements Research	India	*N° of animals*: 24 *Animal breed used*: Murrah *Production*: Reproduction *Aim*: To determine the effects of inorganic chromium on body condition, metabolic responses, lactation performance, and reproductive parameters in periparturient Murrah
Lin	2015	Characterization of the rumen microbial community composition of buffalo breeds consuming diets typical of dairy production systems in Southern China	Animal Feed Science and Technology	China	*N° of animals*: 12 *Animal breed used*: Murrah and Nili-Ravi *Production*: Dairy *Aim*: To investigate the diversity of ruminal microbes in six Murrah and six Nili-Ravi water buffaloes consuming diets typical of those used in Southern China
Garg	2016	Impact of calf nutrition on overall production and productive life of cattle and buffaloes	CABI Reviews	India	This paper is a review article and therefore does not provide specific details regarding animals *Aim*: Discuss the nutritional impact on the growth of calves
Masucci	2016	Effect of group size and maize silage dietary levels on behaviour health, carcass and meat quality of Mediterranean buffaloes	Animal	Italy	*N° of animals*: 28 *Animal breed used*: Mediterranean *Production*: Finishing animals *Aim*: Evaluate the growth performance and behaviour immune response and weight gains of subjects maintained in groups of different sizes
Negesse	2016	Variability in residual feed intake and nutrient utilization in Murrah buffalo heifers	Tropical Animal Health and Production	Ethiopia	*N° of animals*: 18 *Animal breed used*: Murrah *Production*: Growing heifers *Aim*: To determine the residual feed intake
Prusty	2016	Effect of energy and protein levels on nutrient utilization and their requirements in growing Murrah buffaloes	Tropical Animal Health and Production	India	*N° of animals*: 30 *Animal breed used*: Murrah *Production*: Growing buffaloes *Aim*: To determine levels of energy and protein for optimum growth
Sharma	2016	Predicting water intake of lactating riverine buffaloes under tropical climate	Livestock Science	India	*N° of animals*: 18 *Animal breed used*: Murrah *Production*: Milk *Aim*: Investigate water intake under summer conditions
Abo-Zeid	2017	Effects replacing dietary maize grains with increasing levels of sugar beet pulp on rumen fermentation constituents and performance of growing buffalo calves	Animal Feed Science and Technology	Egypt	*N° of animals*: 40 *Animal breed used*: not specified *Production*: Growing buffaloes *Aim*: To evaluate the effects of replacing cracked maize with increasing levels of sugar beet pulp on nutrients digestibility, blood biochemical and rumen fermentation
Hansen	2017	Response of primiparous and multiparous buffaloes to yeast culture supplementation during early and mid-lactation	Animal Nutrition	Denmark	*N° of animals*: 24 *Animal breed used*: not specified *Production*: Milk *Aim*: To investigate effects of yeast supplementation
Sharma	2017	Impact of total dissolved solids in drinking water on nutrient utilisation and growth performance of Murrah buffalo calves	Livestock Science	India	*N° of animals*: 20 *Animal breed used*: Murrah *Production*: Growth of animals *Aim*: Study how total dissolved solids in water can affect animals’ water intake and nutrient utilization
Mahesh and Thakur	2018	Rice gluten meal, an agro-industrial by-product, supports performance attributes in lactating Murrah buffaloes (*Bubalus bubalis*)	Jornal of Cleaner Production	India	*N° of animals*: 18 *Animal breed used*: Murrah *Production*: Milk *Aim*: To investigate the effect of partial substitution of customarily used protein feed peanut cake by rice gluten meal or corn gluten meal
Mor	2018	Effect of ammonia fiber expansion on the available energy content of wheat straw fed to lactating cattle and buffalo in India	Journal of Dairy Science	India	*N° of animals*: 40 *Animal breed used*: Murrah *Production*: Milk *Aim*: To test whether ammonia fiber expansion treatment of wheat straw would increase the energy availability to Murrah
Mudgal	2018	Selenium and copper interaction at supra-nutritional level affecting blood parameters including immune response against P. *multocida* antigen in Murrah buffalo (*Bubalus bubalis*) calves	Journal of Trace Elements in Medicine and Biology	India	*N° of animals*: 10 *Animal breed used*: Murrah *Production*: Growth of animals *Aim*: Evaluate the effect of a diet with supra-nutritional selenium and copper on blood parameters
Nampoothiri	2018	Growth performance, and enteric and manure greenhouse gas emissions from Murrah calves fed diet with different forage to concentrate ratios	Animal Nutrition	India	*N° of animals*: 15 *Animal breed used*: Murrah *Production*: Growing buffaloes *Aim*: Study the effects of different dietary forage to concentrate ratios on performance, and enteric and manure greenhouse gas emissions
Uzun	2018	The inclusion of fresh forage in the lactating buffalo diet affects fatty acid and sensory profile of mozzarella cheese	Journal of Dairy Science	Italy	*N° of animals*: 32 *Animal breed used*: not specified *Production*: Milk *Aim*: To evaluate the fatty acid profile, sensory properties, color, and consumer liking of Mozzarella di Bufala produced under us of fresh sorghum diet
Mudgal	2019	Supra-nutritional copper influences blood parameters including antioxidant markers and immune response in Murrah buffalo (*Bubalus bubalis*) calves	Livestock Science	India	*N° of animals*: 10 *Animal breed used*: Murrah *Production*: Growth of animals *Aim*: Evaluate the effect of supra-nutritional copper on animal growth performance
Orihuela and Mota-Rojas	2020	Weaning strategies to improve productivity and animal welfare in zebu (*Bos indicus*) and water buffaloes (*Bubalus bubalis*)	Journal of Animal Behaviour and Biometeorology	Mexico	*This paper is a review article and therefore does not provide specific details regarding animals* *Aim: Discuss weaning strategies to improve productivity*
Patra	2020	Prediction of nitrogen excretion in buffalo production system using dietary and animal variable	Agricultural Systems	India	*This paper is an analytical article and therefore does not provide specific details regarding animals* *Aim: To develop statistical models to predict nitrogen excretion in buffaloes*
Sharma	2020	Boron supplementation in peripartum Murrah buffaloes: The effect on calcium homeostasis, bone metabolism, endocrine and antioxidant status	Journal of Trace Elements in Medicine and Biology	India	*N° of animals*: 30 *Animal breed used*: Murrah *Production*: Milk animals at peripartum *Aim*: Evaluate the effect of boron supplementation on calcium homeostasis, bone structure, and endocrine status of peripartum buffalo cows
Abdel-Raheem and Hassan	2021	Effects of dietary inclusion of *Moringa oleifera* leaf meal on nutrient digestibility, rumen fermentation, ruminal enzyme activities and growth performance of buffalo calves	Saudi Journal of Biological Science	Saud Arabia	*N° of animals*: 30 *Animal breed used*: not specified *Production*: Growing buffaloes *Aim*: To study the effect of dietary inclusion of moringa leaf meal on nutrient digestibility, rumen fermentation, growth performance
Akhtar	2021	Nitrogen balance, production performance, and plasma metabolites of lactating buffaloes in response to varying dietary protein levels	Tropical Animal Health and Production	Pakistan	*N° of animals*: 6 *Animal breed used*: Nili-Ravi *Production*: Milk *Aim*: To investigate the changes in nitrogen balance, milk production, and plasma metabolites in response to different dietary crude protein supplies
Lakhani	2021	Optimizing fiber and protein levels in diet of lactating Murrah buffaloes to ameliorate heat stress: Effect on physiological status and production performance	Jornal of Thermal Biology	India	*N° of animals*: 362 *Animal breed used*: Murrah *Production*: Milk *Aim*: To assess the outcome of feeding six total mixed rations, differing in neutral detergent fiber and protein content
Lima	2021	Intake, digestibility, and milk yield response in dairy buffaloes fed *Panicum maximum* cv. Mombaça supplemented with seeds of tropical açai palm	Tropical Animal Health and Production	Brazil	*N° of animals*: 5 *Animal breed used*: Half-breed Murrah *Production*: Milk *Aim*: To evaluate the effects of dietary inclusion of açai palm seeds in the supplement on nutrient utilization and milk yield
Salzano	2021	Green feed increases antioxidant and antineoplastic activity of buffalo milk: A global significant livestock	Food Chemistry	Italy	*N° of animals*: 80 *Animal breed used*: Mediterranean *Production*: Milk *Aim*: To determine if the inclusion of green feed further increase milk content of betaines
Abo-Donia	2022	Influence of diets supplemented with naturally protected or unprotected eucalyptus oil on methane production and lactating buffalo productivity	Tropical Animal Health and Production	Egypt	*N° of animals*: 16 *Animal breed used*: not specified *Production*: Milk *Aim*: To investigate the impact of supplementation of eucalyptus oil vs. naturally protect in form of leaves or seed capsules on methane production and performance
Anjum	2022	Effects of maize silage substitution with sugarbeet or citrus pulp ensiled with corncobs on growth performance, digestibility and economic benefits in buffalo calves	Tropical Animal Health and Production	Pakistan	*N° of animals*: 20 *Animal breed used*: Nili-Ravi *Production*: Fattening *Aim*: To investigate the effects of either sugarbeet or citrus pulp ensiled with ground corncobs as replacement of maize silage on finishing animals
Mudgal	2022	Nutraceutical role of supra-nutritional selenium in healthy buffalo (*Bubalus bubalis*) calves	Phosphorus, Sulfur, and Silicon and the Related Elements	India	*N° of animals*: 10 *Animal breed used*: Murrah *Production*: Growth of animals *Aim*: Evaluate the effect of selenium supplementation on buffalo calves’ health
Sharma	2022	Effect of boron supplementation on nutrient utilization and productive performance of peripartum Murrah buffaloes	Biological Trace Elements Research	India	*N° of animals*: 30 *Animal breed used*: Murrah *Production*: Reproduction *Aim*: To determine the effect of boron supplementation on productive performance, apparent nitrogen, and mineral utilization in peripartum
Malafaia	2023	Phosphorus for Cattle and Buffaloes in Brazil: Clinical Signs and Diagnosis of Its Deficiency and Relevance, and Recommended Strategies to Alleviate Issues Observed under Grazing Conditions	Ruminants	Brazil	This paper is a review article and therefore does not provide specific details regarding animals *Aim*: Describe and discuss the phosphorus importance to animal production
Langeh	2024	In-vitro and in-vivo evaluation of dietary cerium supplementation on ruminal fermentation, growth performance and immune status of Murrah buffaloes	Animal Feed Science and Technology	India	This paper involved a prior experiment with in vitro evaluations *N° of animals*: 18 *Animal breed used*: Murrah *Production*: Growth of animals *Aim*: Assess the impact of organic and inorganic cerium supplementation on the growth performance of Murrah buffaloes
Jyani	2024	Supplementation of critical micro-nutrients in peri-parturient dairy buffaloes improves lactation performance	Journal of Trace Elements and Minerals	India	*N° of animals*: 22 *Animal breed used*: Murrah *Production*: Milk *Aim*: Evaluate the effects of micronutrient supplementation (vitamins E, A and niacin, and minerals cobalt, chromium, copper, selenium, and zinc) during the peripartum period on the lactational performance of dairy buffaloes

## Discussion

A greater proportion of the retrieved papers was directly related to milk or dairy production (21), whereas only a few addressed overall animal production applicable to both milk and beef systems. It is important to note that some authors, such as Garg et al. (2016) and Salzano et al. (2021), have described the cultural, nutritional and economic importance of buffalo meat in certain countries (e.g., India, Pakistan, China, and parts of Brazil). This highlights the need for more nutritional studies specifically addressing beef production in buffaloes.

All papers are discussed in sections organized according to DM, CP, energy, neutral detergent fiber (NDF), mineral, and water intakes. Additives and additional considerations are presented at the end of the discussion. None of the included studies fully met the predefined criteria for direct estimation of buffalo nutritional requirements. However, Bartocci et al. (2002) was the study that most closely approached these criteria, and therefore a brief introduction to this study is provided. Nevertheless, it is important to emphasize that this study was based on regression equations derived from data collected in a limited number of farms within a specific geographic region, which limits its representativeness for generalized buffalo nutritional requirement estimation. Paul (2011) also addresses buffalo nutritional requirements; however, as it is a review article, it is considered alongside the other retrieved papers rather than as a primary requirement-estimation study. Although published within the time frame considered in this review (2000–2024), Paul (2011) provides a comprehensive synthesis of earlier research on buffalo nutritional requirements, offering essential background information that complements the more recent studies evaluated here.

In Bartocci et al. (2002), the authors emphasized the cultural importance of buffaloes and analyzed data from 20 farms located south of Rome, Italy. These farms raised the Mediterranean breed and comprised a total of 1,945 animals, including both lactating and dry buffaloes, with body weights (BW) ranging from 570 to 650 kg. The study evaluated animal performance, milk quality, and mozzarella yield as affected by *ad libitum* nutrition.

The nutritional factors evaluated—CP, energy, and DMI—were categorized and correlated with milk production, which ranged from less than 7 to more than 9 L/animal/day, as well as with mozzarella yield. Observations were made across high, intermediate, and low protein and energy levels, with groups constructed based on field conditions. To estimate nutritional requirements, the authors developed regression equations associated with daily milk yield (X) with daily nutrient intake (net energy, CP, and structural and non-structural carbohydrates [Y]):

$$\begin{aligned}&Crude\:protein\:\left(g/day\right)\cr &\:=314.72+(187.35\times\:kg\:milk/animal/day)\end{aligned}$$

$$\begin{aligned}&Milk\:forrage\:unit\:(energy/day)\cr & \:=7.16+(0.66\times kg\:milk/animal/day)\end{aligned}$$

$$\begin{aligned}&Neutral\:detergent\:fiber\:\left(g/day\right)\cr & \:=8864.30+(198.92\times\:kg\:milk/animal/day)\end{aligned}$$

$$\begin{aligned}&Non-structutal\:carbohydrate\:\left(\frac{g}{day}\right)\cr & \:=4762.92+(150.36\times \:kg\:milk/animal/day)\end{aligned}$$

The main limitation of this paper is that the authors did not present an equation for DMI, which impairs practical replication. Moreover, energy requirements were ex-pressed as net energy in milk forage units (Milk FU; Fett-Einheit), which is less practical than expressing them as TDN or metabolizable energy.

### Dry matter intake

Feed intake is commonly expressed as DMI, which is very important in animal production, as it is necessary to design nutritional plans and meet the animals’ nutrient requirements. In this context, Bartocci et al. (2002) observed that animals had an average DMI of 16.75 kg/day. Similarly, Bartocci et al. (2006), working with animals weighing 657–670 kg, reported DMI values ranging from 16.37 to 16.53 kg/animal/day, slightly lower but still very close to those previously reported. These values correspond to an average of 2.48% of BW in dairy buffalo cows.

Lima et al. (2021), working with different levels of açaí palm seed inclusion (0, 198.4, 396.8, and 592.2 g/kg DM) in the concentrate offered to lactating buffaloes kept on pasture, also observed a DMI of approximately 16 kg/day. These values are corroborated by Salzano et al. (2021), who reported a DMI of 16.2 kg/day in dairy buffalo cows receiving green forage in their diet.

Bartocci et al. (2002) also reported that higher-producing animals ingested 17.16 kg DMI/day, which, based on an average BW of 610 kg, corresponds to 2.81% of BW. Based on the available studies, lactating dairy buffaloes generally consumed between 2.48 and 2.81% of BW as DM under the evaluated production conditions. These values are slightly lower than the values reported for cattle at similar production levels (~ 3%) (Paul 2011). However, it is necessary to point out that these values may vary according to breed, diet composition, milk yield, and management system.

For heifers, Gandra et al. (2011) evaluated intake, digestibility, and metabolism in growing buffalo heifers and compared them with Holstein and Nellore heifers, observing differences among genetic groups. Holstein heifers exhibited the greatest intake and digestibility coefficients, whereas Nellore heifers showed the lowest values. Buffalo heifers presented intermediate results, with numerically greater intake and digestibility than Nellore heifers but lower values than Holstein heifers. These differences suggest that caution should be exercised when applying cattle-based nutritional recommendations directly to buffaloes, as species-specific variations in intake and nutrient utilization may influence nutrient requirements. Once this could lead either to increased feed wastage or reduced animal performance. In the same experiment, Gandra et al. (2011) reported a DMI of 8.32 kg/day for buffalo heifers, corresponding to 1.29% of BW, which was lower than that observed for Nellore (7.56 kg/day and 1.54% BW) and Holstein heifers (9.07 kg/day and 1.65% BW) for a similar daily weight gain (0.920 kg/day).

The values reported by Gandra et al. (2011) are lower than those observed by Singh et al. (2014), who reported DMI ranging from 1.97 to 2.12% of BW in heifers weighing 207 ± 9.78 kg. On the other hand, Negesse et al. (2016), evaluating residual feed intake, observed that animals weighing 238 kg had a DMI of 6.6 kg/animal/day, representing 2.78% of BW for an average daily gain of 0.6 kg/day.

In this sense, Tauqir et al. (2011) evaluated growth efficiency in response to different dietary CP and energy levels in male buffaloes averaging 77 kg BW. They reported no differences in DMI (approximately 3% of BW) or average daily gain (390 g/animal/day) among treatments. Similarly, Paul (2011) reported DMI values ranging from 2.2 to 3.15% of BW for growing buffaloes. Prusty et al. (2016) observed a DMI of 6.93 kg DM/animal/day to achieve a gain of approximately 0.600 kg/day, which represented 2.77% of BW in 250 kg animals. Thus, in growing buffalo production systems, DMI generally ranges from 2 to 3% of BW for efficient performance.

### Crude protein

Dry matter intake directly influences the intake of CP, energy, NDF, and minerals. In a meta-analysis of voluntary feed intake and digestibility in four domestic ruminant species (goats, sheep, cattle, and buffaloes), Riaz et al. (2014) observed a direct relationship between DMI and nutrient intake, which, if not properly balanced, could lead to nutritional imbalances. Under field conditions in India, Garg et al. (2016) reported that 71% of dairy buffaloes consumed excess CP and energy. This situation is problematic because unbalanced diets increase feeding costs and nutrient excretion, thereby reducing production profitability (Bartocci et al. 2002; Paul 2011; Garg et al. 2013).

With respect to CP intake, Paul (2011) described CP requirements of 1.11–5.05 g/kg BW^0.75^ for maintenance and 90 g/kg of 6% fat-corrected milk for production. Campanile et al. (2010b), in a review on buffalo protein nutrition and requirements, stated that animals require 3.2 g CP/kg BW^0.75^ for maintenance, a value intermediate to that reported by Paul (2011).

For example, using the 5.05 g and 90 g values, in a scenario of a 600-kg animal producing 9 L of milk, the requirement would be approximately 1,422.2 g CP/day. With a DMI of 2.48% BW (14.88 kg DM), this would correspond to 95.58 g CP/kg DM, or a diet containing approximately 95.58 g CP/kg DM, which is lower than values reported by Bartocci et al. 2002,2006; Chanthakhoun et al. (2012a), and also lower than those recommended by NRC (2001) and INRA (2018) for dairy cows (around 12% CP for efficient production).

Campanile et al. (2010b) further reported that dairy buffaloes require 11–14% CP in the diet, including protein for both maintenance and milk production. However, in their experimental work, CP levels up to 15% of DM stimulated greater milk production. These values are corroborated by Mahesh and Thakur (2018), who observed a CP intake of 1.80 kg/animal/day (14.4% CP on a DM basis) to sustain 10 kg/day of 6% fat-corrected milk. Thus, in the previous example based on Paul (2011), animals would require approximately 2,232 g CP/day rather than 1,422 g, corresponding to 150 g CP/kg DM.

Akhtar et al. (2021) evaluated dietary CP levels of 9.26, 10.0, and 11.4% DM (low, medium, and high protein diets). DMI did not differ among treatments, averaging 14.3 kg/animal/day (lower than values previously discussed for dairy buffaloes). However, as dietary CP increased, nutrient digestibility, milk yield, and milk components (protein, fat, and lactose) also increased, demonstrating improved performance. Nitrogen balance was influenced by dietary CP level, with greater nitrogen excretion at the highest CP level. Although CP increased, NDF and energy concentrations remained similar across treatments, this may have led to an imbalance between protein and energy supply and contributed to increased nitrogen losses (Detmann et al. 2014).

Chanthakhoun et al. (2012a) evaluated diets containing 92, 124, 181, and 219 g CP/kg DM and observed increased digestibility of DM, OM, and NDF beginning at 124 g CP/kg DM. At 92 g CP/kg DM (9.2% CP), microbial population, ammonia concentration, and nutrient digestibility indicated insufficient protein supply for adequate ruminal fermentation. Greater urinary nitrogen excretion was observed at 181 and 219 g CP/kg DM, suggesting that reducing CP intake to 160 g/kg DM could mitigate ammonia emissions and nitrogen losses. Conversely, low dietary protein compromises ruminal microbial growth and fermentation efficiency (Chanthakhoun et al. 2012a, b), reducing milk yield and lactation length (Campanile et al. 2010b). Excessive dietary CP should also be avoided, as it increases nitrogen excretion in blood, milk, and urine (Campanile et al. 2010b) and may cause ammonia toxicity (Chanthakhoun et al. 2012a). Therefore, supplying 150–160 g CP/kg DM (15–16% CP of DM) appears optimal to enhance milk production and lactation duration without compromising health. In high-producing scenarios, these CP levels are similar to or even higher than those suggested by NRC (2001) and INRA (2018) for dairy cows.

Bartocci et al. (2002) reported that animals receiving 134.78 g CP/kg DM showed greater milk production compared with those ingesting 113.96 and 101.40 g CP/kg DM, corresponding to high-, intermediate-, and low-protein groups, respectively. Bartocci et al. (2006) found higher performance with 144 g CP/kg DM and suggested that dietary CP should range between 150 and 160 g/kg DM to maintain adequate body condition throughout lactation.

Adequate nutrition improves production efficiency and increases the likelihood of healthy calves. Proper feeding management during pregnancy contributes to optimal fetal growth, earlier postpartum estrus, and improved milk yield (Garg et al. 2016). However, Garg et al. (2016) did not specify precise nutrient requirements; they only reported that supplementation with 3 kg/day of concentrate during the last trimester increased maternal BW, calf BW, and daily gain. Paul (2011) also highlighted the limited data on nutrient requirements for pregnant buffaloes, breeding bulls, and working animals.

For newborn calves, Garg et al. (2016) recommend colostrum equivalent to 10% of BW within 2 h after birth, followed by an additional 2–3 L within 6 h, consistent with cattle management. Immunoglobulin absorption declines after 6 h. Subsequently, calves should receive at least 4–6 L/day of milk divided into two meals and gradually be introduced to solid feed (starting at 50–100 g/day and increasing to 2% BW of an 18–23% CP concentrate).

Because weaning is a stressful phase, Orihuela et al. (2020) emphasized that animals should be provided with a safe environment and a diet that meets their nutritional requirements. In dairy systems, calves may be fed milk replacers. Garg et al. (2016) noted that replacers for buffalo calves should contain higher CP (24%) and fat (20%) than those formulated for cattle (22% CP and 15% fat).

In beef systems, where weaning occurs later, growing animals are typically fed roughage–concentrate diets. Prusty et al. (2016) evaluated three CP levels combined with two energy levels. Although based on ICAR published in 2013, the final CP and TDN contents were not clearly reported. Initial BW ranged from 201 to 204 kg, and final BW from 293 to 313 kg. CP intake ranged from 267.6 to 332.5 g/day, and energy intake from 20.0 to 22.9 MJ/100 kg BW. Animals fed by high-energy diets achieved greater average daily gain (639 vs. 581 g/day), regardless of CP level. The authors suggested requirements of 6.446 g CP/kg BW^0.75^ for maintenance and 0.463 g CP/kg BW^0.75^ for gain.

These results are consistent with Paul (2011), who suggested 6.0–7.6 g CP/kg BW^0.75^ for maintenance and 0.44–0.51 g CP per g of BW gain. For example, a 250-kg animal gaining 1 kg/day would require approximately 988 g CP/day. With a DMI of 7.5 kg/day, this corresponds to 13.2% CP in the diet. Aasif Shahzad et al. (2011) concluded that 12.2% CP and 2.10 Mcal/kg DM optimized growth in Nili-Ravi calves, whereas Tauqir et al. (2011) reported greater gains with 14.2% CP and 2.24 Mcal/kg DM. Available studies generally reported satisfactory growth performance when dietary CP concentrations ranged from approximately 12.2 to 14.2% DM in growing buffaloes, corroborating with Singh et al. (2014), who reported that 13–15% CP promotes earlier puberty in heifers.

Regarding alternative protein sources, Abdel-Raheem et al. (2021) evaluated partial replacement of soybean meal with Moringa oleifera leaves (15 and 20% replacement). At 15% replacement, DMI and average daily gain increased, despite lower CP and TDN intake. The authors concluded that 15% moringa inclusion may be a viable alternative when soybean meal is costly or scarce.

Tiwari et al. (2001) compared treated and untreated groundnut cake with fish meal and observed no differences in rumen fermentation or blood biochemical parameters, indicating efficient utilization of CP regardless of source. However, animal-derived protein sources such as fish meal are no longer widely recommended in ruminant nutrition due to feed safety concerns (Tretola et al. 2025).

Verma et al. (2000) evaluated a urea–molasses liquid diet combined with two roughage levels. Animals receiving the lower roughage level showed greater digestibility in the first phase. In a subsequent phase, similar daily gains were observed, suggesting metabolic adaptation. However, the evaluation period (120 days per phase) was relatively short. Zia-ul-Hassan et al. (2011) observed no changes in nutrient intake or milk yield when using manure-based silage and varying roughage: concentrate ratios (isonitrogenous at 16% CP), although NDF digestibility increased at 15–25% concentrate inclusion. Kang et al. (2012) similarly reported improved fiber digestibility with in-creased concentrate supply.

Hung et al. (2013) evaluated pellets containing Leucaena leaf powder and urea and concluded that supplementation enhanced intake, nitrogen retention, and microbial protein synthesis. Sultan et al. (2009) reported that decreasing rumen-degradable protein reduced intake and digestibility but increased nitrogen retention at a 55:45 degradable: undegradable ratio. However, other results suggest that 60–65% rumen-degradable protein may optimize intake and digestibility, corroborating Javaid et al. (2011), who identified 66% rumen-degradable protein as optimal.

Finally, nitrogen when not utilized by ruminal microorganisms or animal metabolism is excreted, potentially contaminating the environment (Patra et al. 2020). Patra et al. (2020) developed a model to predict urinary and fecal nitrogen excretion and observed that urinary nitrogen increased as dietary CP increased, particularly when protein and energy were imbalanced. Therefore, understanding energy requirements and sources is essential for optimizing nitrogen utilization in buffalo nutrition.

### Energy

Sabia et al. (2014), evaluating puberty attainment in buffalo heifers kept on pasture or in feedlot, reported that buffaloes generally have a higher DMI relative to BW compared with cattle, although intake was not measured directly in their study. Puberty age did not differ (637 days), but feedlot animals achieved greater BW than pasture animals (432 vs. 373.5 kg). The authors attributed this difference to dietary energy density (0.58–0.60 milk forage units [FU] for pasture vs. 0.80–0.88 for feedlot), suggesting differences in nutrient supply. Nevertheless, buffaloes show high efficiency in energy utilization (both net and gross) (Paul 2011), which may explain the similar hormonal responses observed by Sabia et al. (2014), despite the lower dietary energy in pasture-fed animals.

Reproductive performance was also evaluated by Campanile et al. (2010a), who investigated metabolic homeostasis and ovarian cyclicity under low-energy diets. Animals receiving a diet containing 12.6% CP and 5.8 Milk FU (139% of NRC (2001) energy requirements) achieved greater BW. In contrast, animals fed 86% of energy requirements (with 13.1% CP) maintained BW and reproductive activity but showed reduced oocyte quality. These results confirm that buffaloes utilize energy efficiently, maintaining reproductive function even under restricted energy intake. For growing buffalo heifers, Paul (2011) suggested energy requirements of 1.97 g TDN/g gain and 6.19–9.48 g TDN/kg BW^0.75^ for maintenance, as well as 0.24–0.45 g CP/g gain.

For dairy buffalo cows, Paul (2011) reported maintenance energy requirements similar to those of non-lactating adult ruminants (27–29.78 g TDN/kg BW^0.75^). However, lactating buffaloes require 35.3–49.2 g TDN/kg BW^0.75^, in addition to 406.32 g TDN per kg of 6% fat-corrected milk produced. Bartocci et al. (2002) reported that animals receiving 0.82 Milk FU exhibited the highest efficiency, while Bartocci et al. (2006) observed similar values (0.85–0.94 Milk FU) in multiparous buffaloes throughout lactation.

Bastianetto and Barbosa (2009), in conference proceedings, suggested a correction factor to be multiplied by milk production (based on INRA (2018). It is important to note that their data adjusts milk production to 7% fat content, rather than 6%, and also accounts for milk protein content. Nevertheless, this represents a useful contribution to the field (Table 4).

**Table 4:** Factor of milk fat-correction to dairy buffaloes

Content	% Fat														
% Protein	4.0	4.5	5.0	5.5	6.0	6.5	7.0	7.5	8.0	8.5	9.0	9.5	10	10.5	11
3.0	0.671	0.710	0.749	0.788	0.828	0.867	0.906	0.945	0.984	1.024	1.063	1.102	1.157	1.180	1.219
3.2	0.686	0.726	0.765	0.804	0.843	0.882	0.922	0.961	1.000	1.039	1.078	1.118	1.172	1.196	1.235
3.4	0.702	0.741	0.781	0.820	0.859	0.898	0.937	0.976	1.016	1.055	1.094	1.133	1.188	1.212	1.251
3.6	0.718	0757	0.796	0.835	0.875	0.914	0.953	0.992	1.031	1.071	1.110	1.149	1.204	1.227	1.266
3.8	0.734	0.773	0.812	0.851	0.890	0.929	0.969	1.008	1.047	1.086	1.125	1.165	1.204	1.243	1.282
4.0	0.749	0.788	0.828	0.867	0.906	0.945	0.984	1.024	1.063	1.102	1.141	1.180	1.219	1.259	1.298
4.2	0.765	0.804	0.843	0.882	0.922	0.961	1.000	1.039	1.078	1.118	1.157	1.196	1.235	1.274	1.314
4.4	0.781	0.820	0.859	0.624	0.937	0.976	1.016	1.055	1.094	1.133	1.172	1.212	1.251	1.290	1.329
4.6	0.796	0.867	0.875	0.914	0.953	0.992	1.031	1.071	1.110	1.149	1.188	1.227	1.266	1.306	1.345
4.8	0.812	0.851	0.890	0.929	0.969	1.008	1.047	1.086	1.125	1.165	1.204	1.243	1.282	1.321	1.361
5.0	0.828	0.867	0.906	0.945	0.984	1.024	1.063	1.102	1.141	1.180	1.219	1.259	1.298	1.337	1.376

Adapted from Bastianetto and Barbosa (2009). In it, for example, an animal with an average production of 10 kg, 7,5% fat and 4,2% protein, would be necessary to multiply 10 per 1.039 (10 × 1.039 = 10.39 kg of milk)

However, for fat-corrected milk, a simple equation can be applied by relating the observed fat percentage to the 6% standard described by Paul (2011). Table 5 presents the correction factors based on the following equation:

**Table 5:** Correction factors used in milk buffaloes aiming to standardize to 6% fat content based only on fat content

Variables	% Fat														
	4.0	4.5	5.0	5.5	6.0	6.5	7.0	7.5	8.0	8.5	9.0	9.5	10	10.5	11
Correction factor	0.667	0.750	0.833	0.917	1.000	1.083	1.167	1.250	1.333	1.417	1.500	1.583	1.750	1.750	1.833

Example: A yield of 8 kg of milk with 5.0% fat corresponds to a correction factor of 0.833. Thus, 8 × 0.833 = 6.67 kg of 6% fat-corrected milk

$$\:Correction\:factor=\frac{7.5}{6}=1.250$$

However, it is necessary to emphasize that this correction represents a proportional normalization procedure based on literature standards (Paul 2011), rather than a mechanistic estimation model.

Also, Paul (2011) emphasizes that energy units should be expressed in TDN or metabolizable energy to facilitate comparison across studies. According to INRA (2018), the following conversions apply:

$$\begin{aligned}&Net\:ernergy\:lactation\:\left(NEL\right)\cr &=Milk\:forrage\:unit\times\:7.36,\:\mathrm{and}\end{aligned}$$

$\:NEL\:(MJ/kg)\:=0.1025\times\:TDN\:\left(\%\right)-0.502,$ which needs to be unfolding to:

$$\:TDN\%=\left(NEL+0.502\right)/0.1025$$

Thus, in the Bartocci et al. (2002) scenario (0.82 Milk FU), the TDN requirement corresponds to approximately 63.8% of DM.

From NRC (2001), the conversion from metabolizable energy (ME) to TDN is:

$$\:TDN=\frac{ME}{0.82\times\:4.409}\times 100,$$

where 0.82 is the efficiency of energy utilization and 4.409 is the energy equivalent of 1 kg TDN (Mcal). In the Tauqir et al. (2011) scenario (2.24 Mcal/kg DM), the TDN content corresponds to approximately 62%.

Growing animals require 27.5–52 g of TDN per kg BW^0.75^ for maintenance and 1.44–2.10 g of TDN per g of BW gain (Paul 2011). For example, a 250-kg male buffalo targeting a daily gain of 1 kg and consuming 2.6% of BW would have a DMI of approximately 6.5 kg/day. Based on requirements of 52 g TDN/kg BW^0.75^ for maintenance and 2.1 g TDN/g of gain, this intake would require a dietary TDN concentration of about 80.6%, which is higher than values typically recommended for beef cattle (generally 72–74%). These estimates are supported by Negesse et al. (2016), who evaluated TDN intake through residual feed intake analysis and reported an intake of approximately 5 kg TDN/animal/day, corresponding to about 75% of DMI.

Naik et al. (2009) evaluated the effects of increasing the energy content of adult buffalo diets through supplementation with an inert fat (based on rice bran fatty acid oil combined with calcium hydroxide). The animals received either 300 g of rice bran oil, 200 g of rice bran oil plus 100 g of inert fat, 100 g of rice bran oil plus 200 g of inert fat, or 300 g of inert fat included in the concentrate for a 30-day period. In addition, animals were fed 2 kg of fresh green fodder (*Sorghum bicolor*) and wheat straw, maintaining a forage-to-concentrate ratio of 65:35. The final diets were isonitrogenous (89.7–91.9 g CP/kg DM) and had similar ether extract and total carbohydrate contents in the total mixed ration. No treatment effects were observed on DMI or on the digestibility of DM, CP, OM, or NDF. However, ether extract digestibility increased linearly. The authors concluded that inclusion of 200–300 g of inert fat in straw-based diets increases energy density without compromising DMI or nutrient digestibility.

Similarly, Shelke et al. (2012), also working with protected fat in dairy buffaloes, observed that inert fat affected only ether extract digestibility, leading to increased TDN and metabolizable energy intake in Murrah buffaloes. They also reported increased milk yield with protected fat supplementation, along with reduced milk urea excretion. It is important to note that, in this study, dietary protein was also protected through the use of formaldehyde-treated mustard cake and groundnut oil cake as sources of rumen-protected CP.

The calculations presented above should be considered as approximate comparisons among feeding systems rather than definitive estimates of buffalo energy requirements, as the conversion factors used were originally developed for cattle-based systems and have not been extensively validated for buffaloes. Therefore, the results should be interpreted with caution, considering the potential uncertainty associated with interspecies extrapolation.

### Neutral detergent fiber

Lakahani et al. (2021) investigated dietary fiber levels combined with CP to mitigate the effects of heat stress while maintaining production performance in lactating buffalo cows. The authors evaluated diets containing 30, 34.5, and 37% NDF, with protein levels corresponding to 7.0 and 8.4% of metabolizable protein. Greater DMI was observed when animals were fed the diet containing 34.5% NDF and 8.4% metabolizable protein. This diet also resulted in the highest NDF intake (6.21 kg/day), representing 44.2% of total DMI. In some cases, results obtained with this dietary combination were similar to those observed with other NDF and CP combinations. However, the most notable finding was that milk yield and milk composition (fat, protein, lactose, and total solids) were optimized in animals receiving the diet with 34.5% NDF and 8.4% CP. These findings suggest that 34.5% NDF, when properly balanced with CP, may represent an adequate fiber level for lactating buffaloes under heat stress conditions.

In contrast, Lima et al. (2021) evaluated the inclusion of açaí seed (0, 198.4, 396.8, and 595.2 g/kg DM) in the concentrate offered to lactating buffaloes grazing on *Megathyrsus maximus* cv. Mombasa. Açaí is a common fruit in Northern Brazil, and its seed contains 4.38% CP, 80.9% NDF, and 80.1% DM (Lima et al. 2021). This study is particularly relevant because it evaluated NDF intake relative to BW. The authors observed no treatment effect on NDF intake expressed as % BW, with an average value of 1.48% BW. This value is higher than the 1.2% BW commonly reported for ruminants (Mertens 1987).

Evaluating different inclusion levels of maize silage in diets for fattening buffaloes (ranging from 36 to 10% DM from maize silage, representing higher or lower dietary fiber levels), Masucci et al. (2016) observed no effect of maize silage inclusion on DMI (8.5 kg/day), average daily gain (0.91 kg/day), or carcass weight (251 kg). In another study aiming to reduce maize use in diets for growing buffalo calves, Abo-Zeid et al. (2017) replaced maize with sugar beet pulp—a more fibrous ingredient—at levels of 0, 33.3, 66.7, and 100%. No changes in DMI were observed; however, increasing replacement levels resulted in higher intakes of NDF, CP, and ether extract. Average daily gain increased linearly, ranging from 1.08 to 1.21 kg/day, although final BW was not affected. Dietary NDF intake ranged from 3.50 kg (at 33.3% maize replacement) to 3.99 kg (at 100% replacement), representing approximately 50% of total DMI.

Comparing fresh and conserved forage in dairy buffalo diets, Uzun et al. (2018) observed no effect of roughage type on milk yield (average 9 kg/animal/day), milk fat, protein, lactose content, or sensory characteristics (color, moisture, and acceptance). In contrast, Salzano et al. (2021), working with alfalfa fodder as green forage (included at the level of 30% of DM) compared with a total mixed ration, observed an effect of roughage source on milk fat content. Animals fed green forage showed lower fat content (9.1%), and the milk fatty acid profile was altered. However, milk yield and protein content were not affected. These findings demonstrate the animals’ ability to modulate nutrient use to maintain performance and product quality.

In a different study, Anjum et al. (2022) evaluated the use of ground corncobs as a dry additive in the ensiling of wet sugar beet or citrus pulp to improve silage quality and reduce moisture content. The authors observed that inclusion of 20% ground corncobs increased silage DM content and improved fattening performance of buffalo calves compared with other silage treatments.

Regarding methane production and volatile fatty acid synthesis in relation to fiber intake, Nampoothiri et al. (2018) evaluated changes in roughage-to-concentrate ratios on growth performance, using wheat straw as the roughage source. As the proportion of concentrate increased (from 20% to 60%), dietary NDF content decreased. Neutral detergent fiber digestibility was not affected, remaining at approximately 45%. However, methane pro-duction decreased as the proportion of roughage declined, as expected. A limitation of this study is that NDF intake was not reported.

Fiber digestibility remains a major challenge in ruminant nutrition due to the proportions of cellulose, hemicellulose, and lignin (Lakhani et al. 2021), particularly in countries where crops are not specifically produced for animal feeding (Abdel-Raheem and Hassan 2021). In India, for example, wheat straw is widely used in dairy buffalo diets despite its high lignification. To improve fiber and nutrient digestibility, Mor et al. (2018) evaluated the use of ammonia as an additive to enhance fermentation during wheat straw ensiling. High ammonia fiber expansion (exposure of straw to elevated ammonia levels under high temperature and pressure) increased NDF digestibility from 45.8% in the control treatment to 52.0%. This improvement increased the availability of energy for dairy animals.

The greater NDF digestibility observed in some studies (e.g., Paul (2011); Masucci et al. (2016) may also be associated with characteristics of the buffalo ruminal microbiome. Lin et al. (2015) evaluated the ruminal microbiome of buffaloes raised in China and fed high- or low-concentrate diets (260 vs. 324 g NDF/kg DM, respectively). A greater abundance of fiber-degrading microorganisms—such as *Fibrobacter*, *Paludibacter*, *Ruminobacter*, and *Ruminococcus*—was observed in animals fed low-concentrate diets. These findings indicate that buffalo rumen is well adapted to ferment high-forage diets.

### Mineral

However, although a large number of mineral-related papers were retrieved in this review, the mineral requirements of buffaloes remain poorly defined. Owing to differences in species, experimental objectives and designs, methodologies, and the specific minerals evaluated, the available literature does not allow the establishment of reliable quantitative dietary recommendations for buffaloes. Paul (2011) emphasized that the limited scientific evidence on mineral requirements forces nutritionists to rely on cattle-based recommendations (e.g., NRC (2001). Moreover, the retrieved studies generally evaluated mineral supplementation levels rather than determining actual mineral requirements. Garg et al. (2013), when assessing diet balancing in dairy buffaloes, reported that 65% of the animal’s exhibited calcium and phosphorus deficiencies, highlighting the importance of proper mineral supplementation to improve production efficiency.

Malafaia et al. (2023) conducted a comprehensive review of phosphorus deficiency and its relevance to cattle and buffalo metabolism. They reported that buffaloes are more sensitive than cattle to clinical phosphorus deficiency, although both species exhibit similar clinical signs. Affected animals may show reduced energy status, weight loss, bone fragility, osteophagia (which may predispose animals to botulism), reluctance to move due to pain, recumbency, and even death. However, their study did not establish specific phosphorus requirement values for buffaloes.

Assif Shahzad et al. (2008) evaluated the effects of altering dietary cation (sodium and potassium) and anion (chloride and sulfur) concentrations on DMI, nutrient digestibility, nitrogen balance, and milk yield in lactating buffaloes during summer. Animal BW and initial milk production were not reported. Diets were formulated according to NRC (2001) guidelines. Dietary mineral concentrations (DM basis) ranged from 0.64 to 0.67% phosphorus, 0.71–1.33% calcium, 0.20–0.34% sodium, 0.58–0.60% potassium, 0.28–0.29% magnesium, 0.39–1.61% chloride, and 0.19–0.25% sulfur. Higher DMI and milk yield were observed when animals received 108.16 g/day of phosphorus, 119.9 g/day of calcium, 47.32 g/day of magnesium, 65.91 g/day of chloride, 57.46 g/day of sodium, 270.4 g/day of potassium, and 32.11 g/day of sulfur. Nevertheless, shifts in dietary mineral concentrations also reduced nutrient digestibility, altered serum mineral concentrations, and increased urinary excretion of certain minerals, such as calcium.

Mudgal et al. (2018) and Mudgal et al. (2022) evaluated selenium supplementation in buffalo diets and highlighted its role in metabolic functions related to immunity, growth, disease resistance, and overall performance. Selenium deficiency was associated with impaired health and reproductive performance. Mudgal et al. (2018) also investigated copper supplementation, noting that copper is essential for hemoglobin synthesis, iron metabolism, and normal bone development.

Selenium supplementation alone did not affect serum metabolites or hematological parameters (Mudgal et al. 2018). Similarly, Mudgal et al. (2022) reported no effect on average daily gain, although certain immune responses—such as circulating monocyte counts—were altered. Vitamin concentrations and blood metabolites were unaffected. In a separate study, Mudgal et al. (2019), evaluating copper supplementation alone, observed no changes in most hematological parameters, although neutrophil counts increased and lymphocyte counts decreased.

When selenium and copper were supplemented together, no effect on BW was observed (Mudgal et al. 2019). The authors concluded that combined supplementation improved certain immune responses, although antioxidant status was generally unaffected.

Jyani et al. (2024) investigated micronutrient supplementation in Murrah lactating buffaloes during the peripartum period. The supplement included copper, zinc, cobalt, selenium, chromium, and vitamins A and E, as well as niacin. No differences were observed in DMI or milk yield. Milk composition was affected only on day 68 of lactation, when total solids content changed—possibly associated with peak lactation, as re-ported in cattle (Bondan et al. 2018; Walsh et al. 2024). Although the exact micronutrient levels were not specified, the authors suggested that responses might have been influenced by the basal micronutrient content of dietary ingredients (maize grain, wheat bran, mustard oil cake, and a mineral mixture containing 1% salt during the prepartum period).

Chromium supplementation was evaluated by Deka et al. (2014), who provided in-organic chromium during the periparturient period at levels of 0, 0.5, 1.0, and 1.5 mg/kg DM. Supplementation up to 1.5 mg/kg DM reduced body fat mobilization and alleviated negative energy balance in dairy buffaloes. This finding is particularly relevant in early lactation, when animals must sustain milk production while restoring reproductive function. In a subsequent study, Deka et al. (2015) evaluated chromium supplementation during the peripartum period to determine its effects on reproductive performance, given chromium’s role in enhancing tissue sensitivity to insulin (Lindemann 1996). Supplementation at 1.0 to 1.5 mg/kg DM reduced non-esterified fatty acid concentrations and increased blood glucose levels, promoting a more favorable energy balance. The authors concluded that chromium supplementation may help prevent reproductive disorders. However, limitations include the small number of animals per treatment (six animals) and the absence of direct measurements of reproductive resumption.

Another trace mineral studied in dairy buffalo nutrition was boron (Sharma et al. 2020). Animals received boron supplementation from 45 days prepartum to 120 days postpartum. Boron was reported to influence calcium metabolism during the transition period, which is closely related to milk fever and calcium–phosphorus balance. Supplementation up to 400 ppm altered serum concentrations of vitamin D₃, calcium, phosphorus, and magnesium, although immune and metabolic parameters were largely unaffected. The authors recommended supplementation at 200 ppm. In a subsequent study, Sharma et al. (2022) reported that boron supplementation during the prepartum (30 days) and postpartum (90 days) periods decreased blood concentrations of non-esterified fatty acids. DMI, nutrient digestibility, and nitrogen balance were not affected. Before calving, magnesium and zinc absorption coefficients increased, whereas postpartum, calcium, magnesium, and zinc absorption were enhanced. These minerals are closely associated with structural integrity and energy metabolism (Muiño et al. 2024).

More recently, Langeh et al. (2024) evaluated the use of rare earth elements in buffalo nutrition, specifically cerium, in both in vitro and in vivo experiments to assess its potential to modulate ruminal fermentation. Two sources were tested: cerium oxide (inorganic) and cerium acetate (organic). Supplementation with either form at 300 mg/kg of diet altered fermentation patterns, increased propionate production, and enhanced certain immune responses. The DMI, average daily gain, and nutrient digestibility were not affected.

### Additives

Regarding the use of additives in buffalo nutrition, Khattab et al. (2010) conducted an interesting study evaluating natural, biological, and chemical additives—such as berseem clover, rice straw, yeast culture, chamomile flowers, fenugreek seeds, garlic seeds, sodium acetate, and sodium succinate—used individually or in combination in dairy buffaloes. The authors observed that the combination of chamomile, sodium acetate, and sodium succinate resulted in the greatest improvements in nutrient digestibility, milk yield, and milk composition. No deleterious effects were reported, indicating that the use of specific additives in dairy buffalo diets can enhance animal productivity.

Hansen et al. (2017) evaluated supplementation with a Saccharomyces cerevisiae–based yeast during early and mid-lactation in Egyptian dairy buffaloes. Yeast was sup-plied at 10 g/animal/day. The authors observed increases in DM and CP intake, as well as improvements in DM, CP, and NDF digestibility. In addition, milk yield, lactation persistency, and milk fat and protein contents were enhanced.

Paul et al. (2004) evaluated the use of a non-indigenous anaerobic fungal strain (*Piromyces* sp. FNG5) in buffalo nutrition to determine whether its inclusion would enhance fiber digestibility. The diet contained 262 g/kg DM of NDF in the concentrate and 855 g/kg DM of NDF in wheat straw. Inclusion of the fungal additive did not affect DMI. However, DM, organic matter, and NDF digestibility increased, demonstrating that fungal additives can improve the nutritional value of high-fiber diets.

Similarly, Sarwar et al. (2006) investigated strategies to improve the digestibility of wheat straw, a high-fiber, low-quality feed. Different levels of urea (0, 2, and 4%) and molasses (2 and 4%) were combined with 30% cattle manure, and the mixture was fermented for 20, 30, or 40 days. No increases were observed in the apparent digestibility of DM, organic matter, CP, or NDF. However, the inclusion of 4% urea and 4% molasses increased the CP content and reduced the fiber content of the treated material, allowing it to replace concentrate at levels of up to 35% of the ration in growing buffalo calves.

Abo-Donia et al. (2022) evaluated the inclusion of protected or unprotected eucalyptus oil in lactating Egyptian buffaloes, aiming to reduce methane production through modulation of ruminal metabolism. According to the authors, eucalyptus oil can alter ruminal biohydrogenation, thereby reducing methane emissions. The animals received 200 g of protected oil or eucalyptus in its natural forms (leaves or green seed capsules). Results showed an increase in ruminal pH, alterations in volatile fatty acid synthesis, and reduced methane production in animals receiving eucalyptus, regardless of the form. However, DM, CP, NDF, and TDN intakes were negatively affected by eucalyptus supplementation, which may limit its practical application in dairy buffalo diets.

### Water intake

Regarding water intake, Sharma et al. (2016) investigated water consumption in lactating Murrah buffaloes and proposed a predictive model based on DMI, milk yield, dietary moisture, and climatic variables. They concluded that water intake in lactating buffaloes is influenced simultaneously by productivity, feed characteristics, and environmental factors. To estimate water consumption, the authors proposed the following equation:

$$\begin{aligned}DWI&=\:-43.15-\left(0.22\times FWI\right)+\left(0.99\times MY\right)\cr & \quad+\left(1.44\times DMI\right)+(0.56\times T\:^\circ\:C)\cr & \quad+\left(0.80\times \%DM\right),\end{aligned}$$

where DWI = drinking water intake (L/day); FWI = feed water intake (determined as DMI × moisture content of diet); MY = milk yield (kg/day); DMI = dry matter intake (kg/day); T = maximum daily temperature (°C); and %DM = dry matter content of total ration.

Based on this model, the authors observed that water intake in Murrah lactating buffaloes ranged from 4.65 to 5.27 L/kg of DMI, which is consistent with Paul (2011), who reported a direct relationship with DMI, with values between 5.2 and 5.5 L/kg of DMI. These results are also corroborated by Mahesh and Thakur (2018), which evaluated the replacement of protein feed peanut cake by rice or corn gluten meals and observed a ratio of 5.85 L of water intake/kg of DMI.

However, one important aspect to consider is water quality. Sharma et al. (2017), when evaluating the effect of total dissolved solids on water intake, DMI, and nutrient utilization, reported a reduction in DMI and average daily gain, along with an increase in urinary excretion (aimed at reducing excess salt and leading to dehydration) as the dissolved salt concentration increased. These findings indicate that high water salinity (> 4,500 mg total dissolved solids/L) negatively affects production efficiency in buffaloes.

### Limitations of the review

This review has several limitations that should be considered when interpreting the results. First, there is a substantial lack of published information regarding buffalo nutritional requirements, particularly for DMI, nitrogen, energy, fiber, and mineral nutrition, as well as for different production contexts such as growth, milk production, reproduction, and meat systems. This scarcity of data, combined with the heterogeneity among studies in terms of breeds, animal categories, experimental designs, and production systems, limits the possibility of synthesizing results into unified nutritional recommendations. As a consequence, meta-analysis or quantitative data integration was not feasible, and the findings are presented in a structured narrative and descriptive manner.

Second, although all efforts were made to ensure comprehensive literature retrieval, limitations related to database indexing and search consistency were identified. For example, some relevant publications were inconsistently retrieved across databases, such as Campanile et al. (2010b), which was not consistently identified in the CABI Digital Library search but was retrieved via Google Scholar. In addition, the ICAR published in 2013, a highly relevant reference on cattle and buffalo nutrient requirements cited in several studies (e.g., Prusty et al. 2016; Sharma et al. 2016), is not freely accessible, which may limit its inclusion in systematic searches.

Finally, due to these constraints, particularly the limited number of studies and lack of methodological standardization, this review does not aim to generate definitive nutritional requirement tables. Instead, its main contribution is to synthesize the current state of knowledge, highlight existing gaps, and support the prioritization of future research in buffalo nutrition.

## Conclusions

There is a very limited number of papers published in English, open-access, and indexed journals on buffalo nutritional requirements over the past 25 years. Only 58 papers were retrieved, and just one directly evaluated nutrient requirements (dry matter, protein, and net energy). Another paper addressed the topic, but it was a re-view.

Most of the retrieved studies focused on dairy production, while aspects such as reproduction, growth, and beef production were largely overlooked—often with additional limitations related to the breeds evaluated. Therefore, there is an urgent need for more research on buffalo nutritional requirements to avoid malnutrition and prevent the inappropriate application of nutritional recommendations derived from other species.

## Data Availability

The data that support the findings of this study are available from the corresponding author, upon reasonable request.
